# Unraveling the microbiome–aroma Nexus: a metagenomic and volatile compound analysis of Yunnan cigars

**DOI:** 10.3389/fmicb.2025.1597501

**Published:** 2025-07-09

**Authors:** Jing Pan, Ming-Da Huang, Jin Wang, Jian-Xiong Zhao, Bin Yang, Hong-Hui Yang, Jin-Sheng Huang, Yu-Long Su, Xue-Ru Song, Wei-Guang Wang, Ling-Duo Bu

**Affiliations:** ^1^Key Laboratory of Natural Products Synthetic Biology of Ethnic Medicinal Endophytes, State Ethnic Affairs Commission, Key Laboratory of Chemistry in Ethnic Medicinal Resources, Ministry of Education, Yunnan Minzu University, Kunming, China; ^2^Yunnan Key Laboratory of Tobacco Chemistry, China Tobacco Yunnan Industrial Co., Ltd., Kunming, China; ^3^Yunnan Tobacco Company, Lincang Branch, Lincang, China; ^4^Yunnan Tobacco Company, Yuxi Branch, Yuxi, China

**Keywords:** cigar, metagenomic, volatile organic compounds, microbial community, microbial metabolism, microbiome

## Abstract

**Introduction:**

Understanding how microbial communities influence aroma profiles is critical to improving cigar quality. However, comparative studies examining the microbiome–aroma nexus across major cigar-producing regions remain limited.

**Methods:**

We integrated high-throughput metagenomic sequencing with volatile organic compound (VOC) profiling to investigate microbial community structure and aroma compounds in four Yunnan cigars and two Cuban cigars.

**Results:**

*Bacillus* spp. was consistently dominant across all samples, while Yunnan cigars exhibited higher microbial diversity. A total of 121 VOCs were detected, with nicotine, ylangene, *δ*-elemene, (R,S)-anatabine, and phenethyl alcohol identified as key aroma components. Nicotine, accounting for 29.7–55.0% of total VOC content, was positively correlated with *Enterobacter* and *Escherichia*, and negatively with *Corynebacterium* and *Brachybacterium*. Ylangene showed strong positive associations with *Brachybacterium* and *Yaniella*. After FDR correction, 26 differential VOCs were identified across cigar groups. KEGG pathway analysis revealed functional enrichment in carbohydrate, amino acid, and lipid metabolism. Principal component analysis indicated that the aroma complexity of certain Yunnan cigars, particularly YX4, approached that of Cuban cigars.

**Discussion:**

Our findings demonstrate that region-specific fermentation microbiota are intricately linked to the production of key VOCs. This work provides a scientific framework for optimizing cigar fermentation through microbial regulation and supports the potential for targeted microbial inoculation to enhance sensory quality and global competitiveness.

## 1 Introduction

Cigars are globally recognized as high-value cash crops, appreciated for their cultural heritage, complex flavor profiles, and high quality (Xing et al., 2021; Koszowski et al., 2018; Allem et al., 2019). Among the leading production regions, Cuba and Yunnan (China) have developed distinctive cigar products shaped by local environmental conditions and fermentation practices. Cuban cigars are renowned for their meticulous craftsmanship and controlled fermentations, often involving selected microbial inoculants to enhance flavor development (Viola et al., 2016). Yunnan cigars rely on spontaneous fermentation mediated by indigenous microbial communities and benefit from the region’s subtropical monsoon climate and traditional processing techniques (Jing et al., 2022; Chen et al., 2020).

As global demand for premium cigars continues to grow—projected to reach US $70.8 billion by 2032 with a CAGR of 4.6% —understanding the microbial and chemical determinants of cigar quality is increasingly important. As a prominent representative of traditional cigar markets, Cuba’s leading cigar manufacturer, Habanos S. A., achieved sales of USD 827 million in 2024, marking a 16% year-over-year increase (Allied Market Research, 2023). In China, the domestic cigar market is expanding even more rapidly, with a CAGR exceeding 7%. Yunnan cigars, primarily sold to cigarette manufacturers in provinces such as Shandong, Hubei, and Anhui, are in short supply, with 2024 sales at approximately 200 million yuan and an annual growth rate of 5–6% (RenrenDoc, 2025). These trends highlight Yunnan’s potential for both domestic dominance and global market expansion.

Fermentation is a key determinant of cigar quality, driving complex biochemical transformations through microbial metabolism, enzymatic activity, and interactions with environmental parameters such as temperature, humidity, and soil composition (Chattopadhyay et al., 2021; Zhang et al., 2024). These processes result in the generation of volatile organic compounds (VOCs), which contribute to characteristic aromas including nutty, floral, and coffee-like notes (Morris and Fiala, 2015; Krüsemann et al., 2018; Zheng et al., 2022). The metabolic activities of the microbial community encompass fatty acid and lipid biosynthesis, chlorogenic acid breakdown, carbohydrate degradation, protein catabolism, as well as amino acid and aromatic compound synthesis (Banožić et al., 2020; Su et al., 2024). Aldehydes and ketones, characterized by their carbonyl groups, contribute to tobacco’s distinctive aroma; esters add floral, fruity, and sweet notes; pyrazines impart nutty and roasted flavors; and furan compounds introduce a caramel-like scent (Chen et al., 2008; Villemur et al., 2009; Si et al., 2023).

Microbial communities associated with tobacco leaves—primarily bacteria and fungi—play pivotal roles in shaping these VOCs (Wu et al., 2023; Zhang et al., 2023). Bacterial genera frequently identified in tobacco include *Bacillus*, *Sphingomonas*, *Stenotrophomonas*, *Erwinia*, *Pantoea*, *Lactococcus*, *Clostridium*, *Staphylococcus*, *Enterobacter*, and *Paenibacillus* (Huang et al., 2010; Su et al., 2011). For instance, *Pseudomonas* efficiently degrades nicotine, while *Bacillus* breaks down lignin and carotene by secreting specific enzymes, yielding small aromatic compounds (Ye et al., 2017; Chopyk et al., 2017). Meanwhile, fungal such as *Aspergillus*, *Penicillium*, *Alternaria*, *Chaetomium*, *Cladosporium*, *Rhizopus*, *Fusarium*, *Trichoderma*, and *Monascus* are also common (Chen H. et al., 2018; Chen S. et al., 2018).

Environmental conditions, particularly temperature, humidity, and altitude, exert strong influences on microbial community composition and activity. For example, elevated temperatures in tropical and subtropical regions like Cuba and Yunnan promote the proliferation of thermophilic and mesophilic microorganisms that degrade carotenoids and terpenoids, leading to the formation of key aroma compounds such as ionone and dihydroactinidiolide (English et al., 1967; Maldonado-Robledo et al., 2003). High humidity favors the growth of fungi such as Aspergillus and Penicillium, enhancing floral and earthy VOCs (Liu et al., 2015; Wu et al., 2022). Additionally, altitude influences microbial diversity by altering soil pH and nutrient availability, while anthropogenic factors such as heavy metal pollution can suppress microbial richness and shift community structure (Ye et al., 2024; Liu et al., 2020).

Advances in omics technologies, including 16S rRNA gene sequencing, shotgun metagenomics, and metabolomics, have enabled comprehensive profiling of biological components such as microbial taxa, functional genes, protein expressions, and metabolic signatures within natural ecosystems (Zhang et al., 2021; Romdhane et al., 2022). These approaches have been increasingly applied to tobacco-related studies (Liu et al., 2021; Ye et al., 2021; Zhou et al., 2021), revealing the influence of geographic origin and leaf variety on VOCs (Tang et al., 2020; Yan et al., 2022; Zhengfeng et al., 2024). A thorough investigation of these aroma compounds not only elucidates how they differ across various cigar types but also provides a scientific framework for quality assessment (Wang et al., 2024).

However, systematic comparisons between major cigar-producing regions, such as Cuba and Yunnan, remain limited, particularly in elucidating how regional fermentation microbiota shape the aromatic profiles of cigars. We hypothesize that distinct microbial communities may be shaped by the unique environmental conditions of Yunnan and Cuba, drive differences in VOCs that define their respective cigar aromas, potentially enabling Yunnan cigars to achieve aromatic profiles comparable to those of Cuban cigars. To evaluate this hypothesis, we employed high-throughput metagenomic sequencing and GC–MS to analyze the microbial compositions and volatile profiles of cigars from both regions, aiming to elucidate the role of microbial communities in shaping region-specific aroma profiles. As the first integrated microbiome–metabolome comparison of Yunnan and Cuban cigars, our work provides mechanistic insights into aroma formation and offers a scientific basis for improving cigar quality through microbial modulation.

## 2 Materials and methods

### 2.1 Experimental materials

In this study, we collected four Yunnan cigars labeled YX1 (Huaning, 24°11′N, 102°55′E; altitude about 1,200 m), YX2 (Ganzhuang, 23°45′N, 102°2′E; 700–800 m), YX3 (Xinping, 24°40′N, 101°59′E; 500–550 m), and YX4 (Chengjiang, 24°40′N, 102°54′E; 1,650 m), along with two Cuban cigars labeled YX5 (Vuelta Abajo, 22°24′N, 83°42′W; 150 m) and YX6 (Vuelta Abajo, 22°24′N, 83°42′W; 100–200 m) for analysis. All the cigars were subjected to an identical fermentation process in 2020 and subsequently aged for 2 years before reaching their final form. The fermentation experiments were carried out with pest control, sorting, rehumidification, balancing, stacking, fermenting, repiling (five times), and unstacking. Repiling occurred immediately when the temperature reached 40–45°C. After that, the cigars were aged for 2 years under controlled conditions of 20°C and 70% humidity (Zhang et al., 2025a). The samples were selected to represent different varieties and geographical origins, as detailed in Table 1 Information of cigars. For each sample, we weighed out 6.0 g, divided it into six portions, and immediately froze the portions in sterile centrifuge tubes using liquid nitrogen. To maintain sample integrity, three portions from each group were stored at −20°C for GC–MS analysis while the remaining three were stored at −80°C for DNA extraction, in accordance with the requirements of each analytical procedure.

**Table 1:** Information of cigars.

Place	Sample	Denomination of origin	Altitude
Yunnan	YX1	Huaning (24°11′N, 102°55′E)	About 1,200 m
	YX2	Ganzhuang (23°45′N, 102°2′E)	700–800 m
	YX3	Xinping (24°40′N, 101°59′E)	500–550 m
	YX4	Chengjiang (24°40′N, 102°54′E)	About 1,650 m
Cuba	YX5	Vuelta Abajo (22°24′N, 83°42′W) Vuelta Abajo	About 150 m
	YX6	(22°24′N, 83°42′W)	100–200 m

### 2.2 Experimental methods

#### 2.2.1 Metagenomic sequencing

DNA extraction, library construction, and sequencing were carried out at Beijing Novogene Co., Ltd., with each sample processed in triplicate. Approximately 1 μg of genomic DNA from each sample was randomly sheared into ~350 bp fragments using a Covaris ultrasonic disruptor (LE220R-plus, United States). Subsequent steps included end repair, addition of A-tails, adapter ligation, purification, and PCR amplification. The integrity and insert sizes of the resulting libraries were assessed by AATI analysis. Once the insert length met the requirements, the libraries were precisely quantified via Q-PCR to ensure an effective concentration above 3 nM. Libraries that passed quality control were then pooled based on effective concentration and target data yield, followed by PE150 sequencing (Illumina Novaseq 6,000, Illumina, San Diego, CA, United States).

#### 2.2.2 Bioinformatic analysis

Raw Illumina data were preprocessed with Fastp,^1^ yielding clean reads for downstream analyses. We discarded paired reads if they contained adapter contamination, had more than 10% ambiguous nucleotides, or showed over 50% low-quality bases (Q < 5). The clean data were assembled using MEGAHIT with the meta-large parameter settings (−-end-to-end, −-sensitive, -I 200, −X 400). Scaffold sequences were broken at “N” junctions to generate contiguous sequences (scaftigs) without the presence of “N”.

Using default parameters, MetaGeneMark^2^ predicted open reading frames (ORFs) on scaftigs ≥ 500 bp for each sample (Karlsson et al., 2012; Oh et al., 2014; Qin et al., 2014). Any predicted segments under 100 nt were removed (Sunagawa et al., 2015). Redundant genes were clustered using CD-HIT^3^ with the parameters -c 0.95, −G 0, -aS 0.9, −g 1, and -d 0, producing a non-redundant initial gene catalog (Fu et al., 2012). Clean reads from each sample were aligned to this catalog via Bowtie2 (−-end-to-end, −-sensitive, -I 200, −X 400) to obtain gene read counts; genes with ≤ 2 reads in each sample were removed, resulting in the final gene set (Unigenes) for subsequent analyses (Li et al., 2014). Gene abundance was calculated based on the number of aligned reads (r) and the gene length (L) (Villar et al., 2015).

#### 2.2.3 Volatile flavor compounds analysis

VOCs in cigars were analyzed via headspace solid-phase microextraction–gas chromatography–mass spectrometry (HS-SPME-GC–MS). Each 3.0 g sample was finely chopped, placed into three 20 mL headspace vials, ensuring biological replication. Each vail was spiked with 4 μL of a 5.0 μg/μL deuterated naphthalene (Naphthalene-d8) solution as an internal standard. The vials were sealed with silicone septa and incubated at 80°C for 20 min. An 85 μm CAR/PDMS fiber (MPS-Robotic, Gerstel, Germany) was then used to extract volatile compounds for 5 min at 80°C, followed by desorption at 250°C for 5 min.

Subsequent separation and identification of volatile flavor compounds were carried out on an Agilent 7890B gas chromatograph (Santa Clara, CA) equipped with a 5977B mass spectrometer and a DB-5MS capillary column (60 m × 0.25 mm × 0.25 μm film thickness). High-purity helium served as the carrier gas at a constant flow rate of 1 mL/min, with an injection port temperature of 250°C. The oven temperature was initially held at 40°C for 1 min, ramped at 5°C/min to 220°C, and maintained for 3 min. The transfer line and ion source were set to 270°C and 230°C, respectively, under electron impact (EI) ionization at 70 eV in full-scan mode (m/z 30–550). Compound identification was based on comparisons with authentic standards (when available) and the NIST 17 library.^4^ For semi-quantitative analysis, peak areas of individual compounds were normalized against the internal standard peak area (Naphthalene-d8) to determine relative concentrations (Zhang et al., 2024).

### 2.3 Data mining and statistical analysis

All experiments were conducted with at least three replicates per condition, and results are presented as mean values to ensure reliability and reproducibility. Heatmaps, principal component analysis (PCA), boxplots, and multiple comparisons were generated in Origin 2024 and GraphPad Prism 8.0.1. Additionally, we used Spearman’s correlation coefficients to examine the relationships between representative microbes and core VOCs, and performed network analyses in PyCharm Community Edition 2023.2.3. Kruskal-Wallis and Wilcoxon rank sum test are calculated in R (v4.2.2).

## 3 Results

### 3.1 Overview of the microbial community

**Figure 1:**
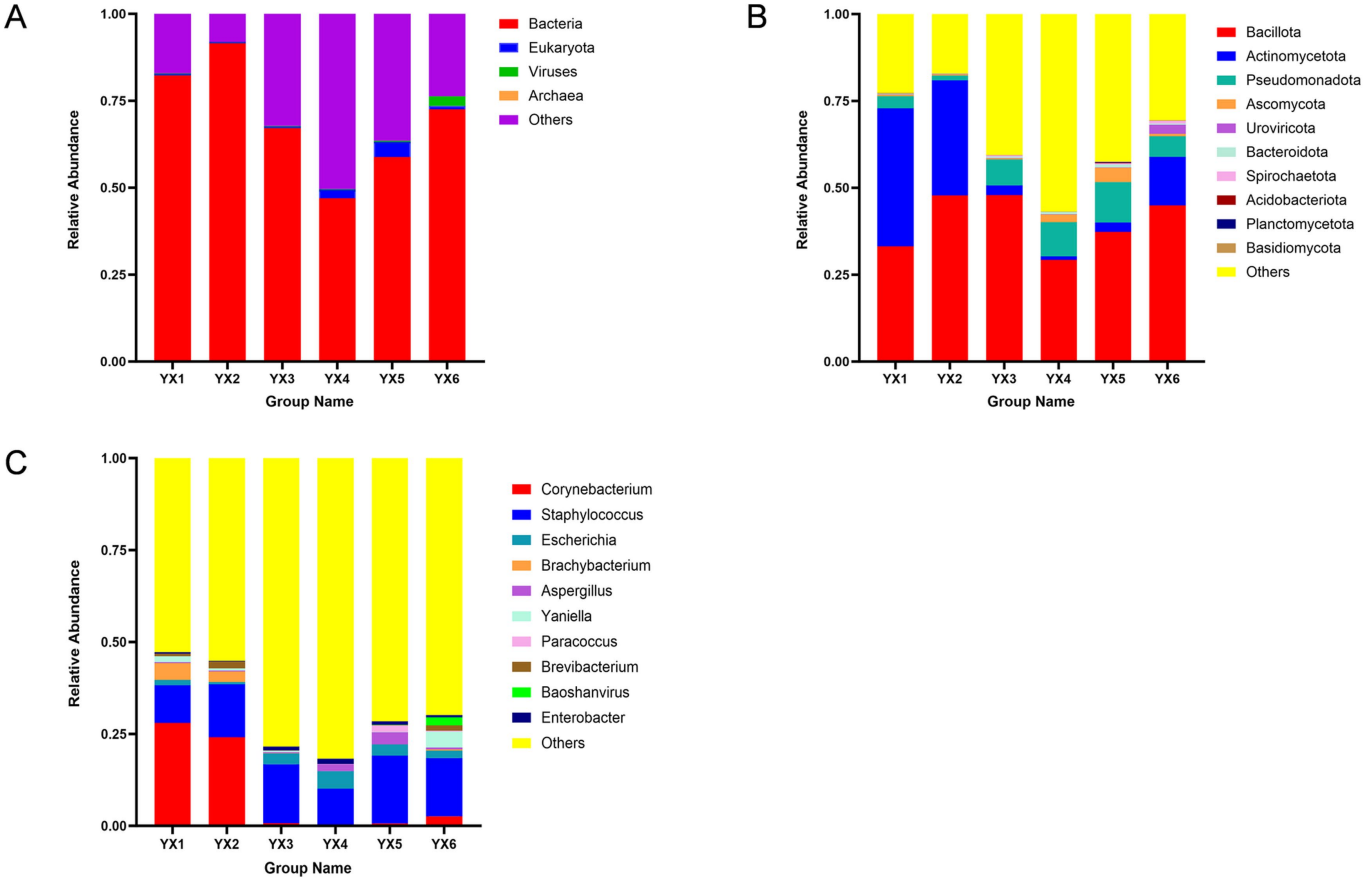
Microbial community composition of cigars at kindom (**A**), phylum (**B**) and genus (**C**) levels.

After quality filtering, each of the 18 samples yielded between 67,247,870 and 99,945,732 high-quality sequences. Coverage analysis indicated that this sequencing depth was sufficient for saturation, ensuring robust capture of microbial diversity. As illustrated in Figure 1A, taxonomic analysis revealed that bacterial communities dominated the microbial composition in all samples, consistently accounting for close to or exceeding 50% of the total. This finding underscores the central role of bacteria in colonizing both Yunnan and Cuban cigars.

We classified microbial groups at two taxonomic levels, the top 10 phyla and genera by relative abundance were illustrated in the figures. At the phylum level, taxa with relative abundances above 0.5% were defined as dominant phyla, while at the genus level, those above 1% were considered dominant genera (Yao et al., 2024). At the phylum level (Figure 1B), *Bacillota*, *Actinomycetota*, and *Pseudomonadota* collectively accounted for 30.25–80.92% of total abundance. *Bacteroidea* also exhibited high abundance across all samples, suggesting a notable influence on the overall microbial composition. At the genus level (Figure 1C), *Staphylococcus* emerged as a dominant taxon, with high abundance in all six samples. *Corynebacterium* was particularly abundant in Yunnan cigars YX1 and YX2, making it a key genus in those two samples.

Despite these shared characteristics, microbial abundances varied substantially by geographic origin. For instance, *Actinomycetota* was significantly more abundant in YX1, YX2, and YX6 than in YX3, YX4, and YX5. Although *Bacillota* remained the most prevalent phylum overall, *Pseudomonadota* also had a noteworthy presence in YX3, YX4, and YX5. At the genus level, *Paracoccus* was found only in YX5, while *Baoshanvirus* appeared exclusively in YX6. In general, Yunnan cigars exhibit *Bacillus* dominance.

**Figure 2:**
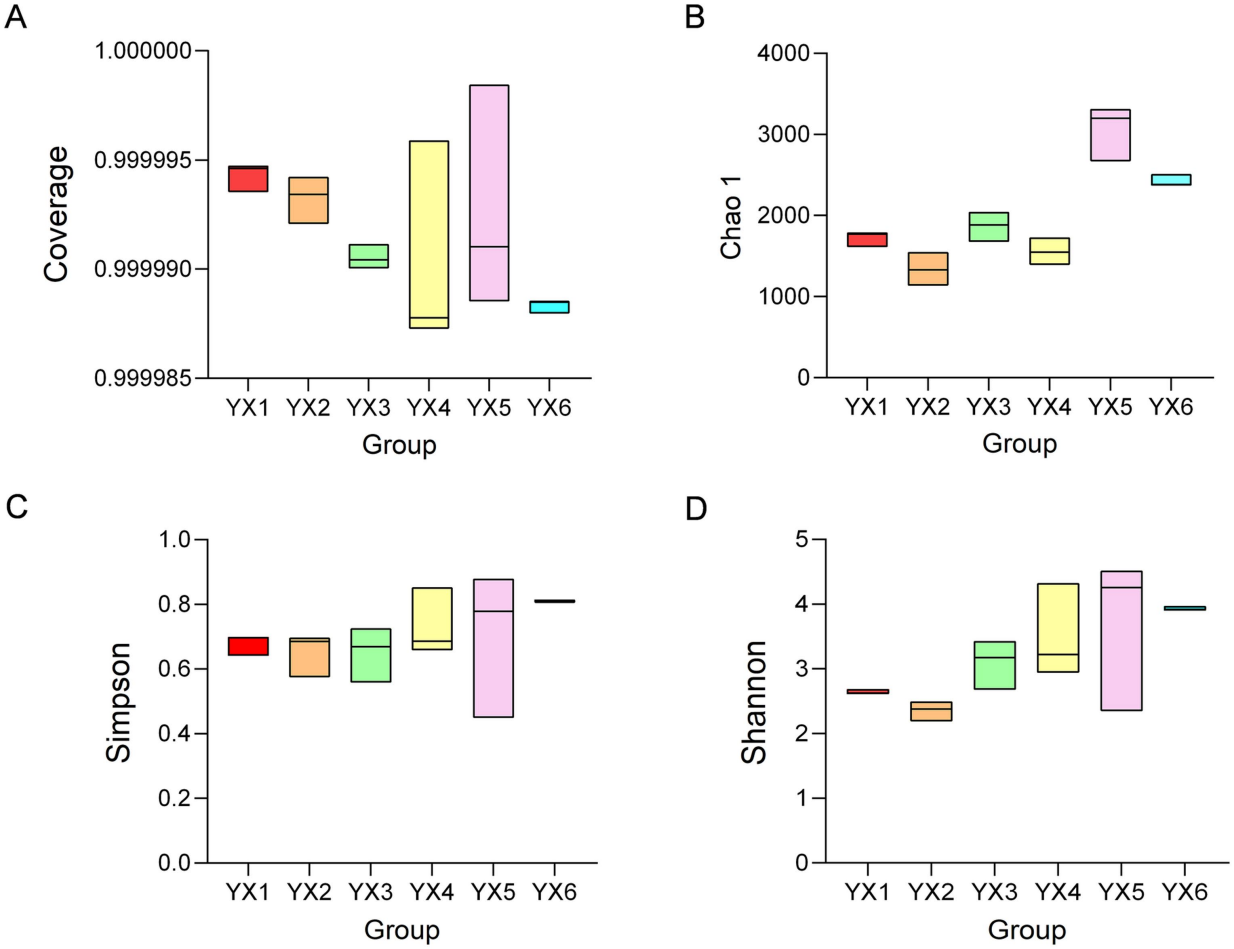
Alpha Indices. Coverage **(A)**, Chao1 index **(B)**, Shannon index **(C)**, Simpson index **(D)**.

An alpha-diversity analysis was performed on the six cigar types. Microbial coverage for all samples approached 1 (Figure 2A), indicating high sequencing coverage and reliable sampling to reflect the cigar-associated microbial communities. The Chao1 index reflects community richness, whereas the Shannon and Simpson indices measure community diversity. The Chao1 index showed that Cuban cigars displayed higher bacterial richness than Yunnan cigars (Figure 2B), and YX5 also exhibited high species richness and evenness. Moreover, Yunnan cigars YX3 and YX4 are different from YX1 and YX2, demonstrated enhanced microbial richness and evenness (Figures 2C,D). Notably, YX4 exhibited clustering patterns more closely aligned with YX5.

**Figure 3:**
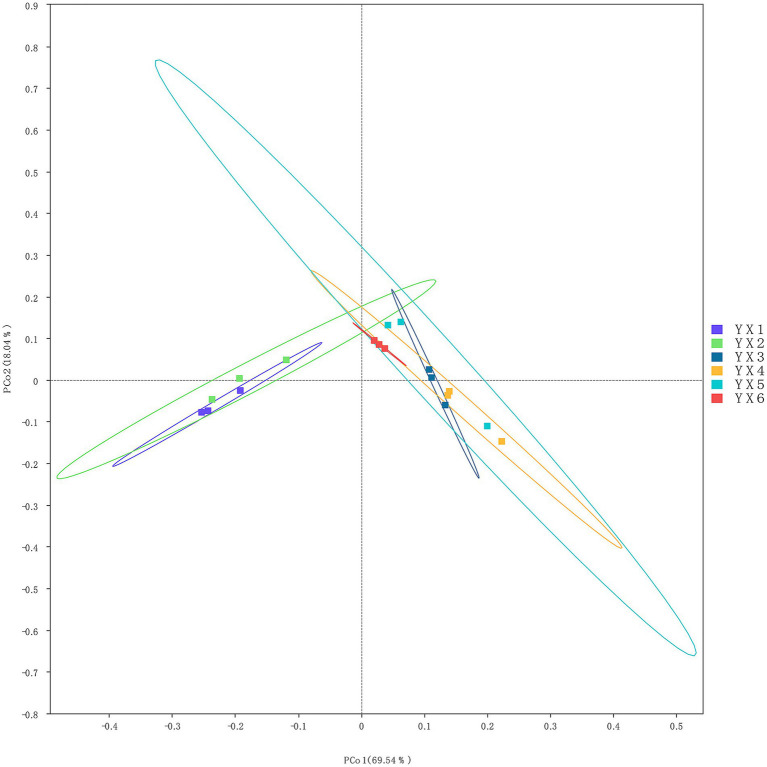
Beta diversity analysis of six cigar samples.

Then, we use the principal coordinate analysis (PCoA) plot illustrates the variation in microbial community compositions across the six cigar samples (YX1–YX6) (Figure 3). The first two principal coordinates, PCoA 1 and PCoA 2, account for 69.54 and 18.04% of the total variance, respectively, capturing the majority of the dataset’s structural variability. Samples YX1 and YX2 are closely clustered in the left quadrant, signifying a high degree of similarity in their microbial profiles. Conversely, four samples clustered in the right quadrant, which means the composition of these microorganisms is significantly different from that of YX1 and YX2. Notably, YX5 exhibits greater dispersion, indicative of increased diversity in their microbial compositions. And the overlap observed between YX4 and YX6 suggests that the fermentation modifications applied to YX4 contribute to the convergence of its profile with that of premium Cuban cigars.

### 3.2 Predicted metabolic functions of microbial communities in cigars

**Figure 4:**
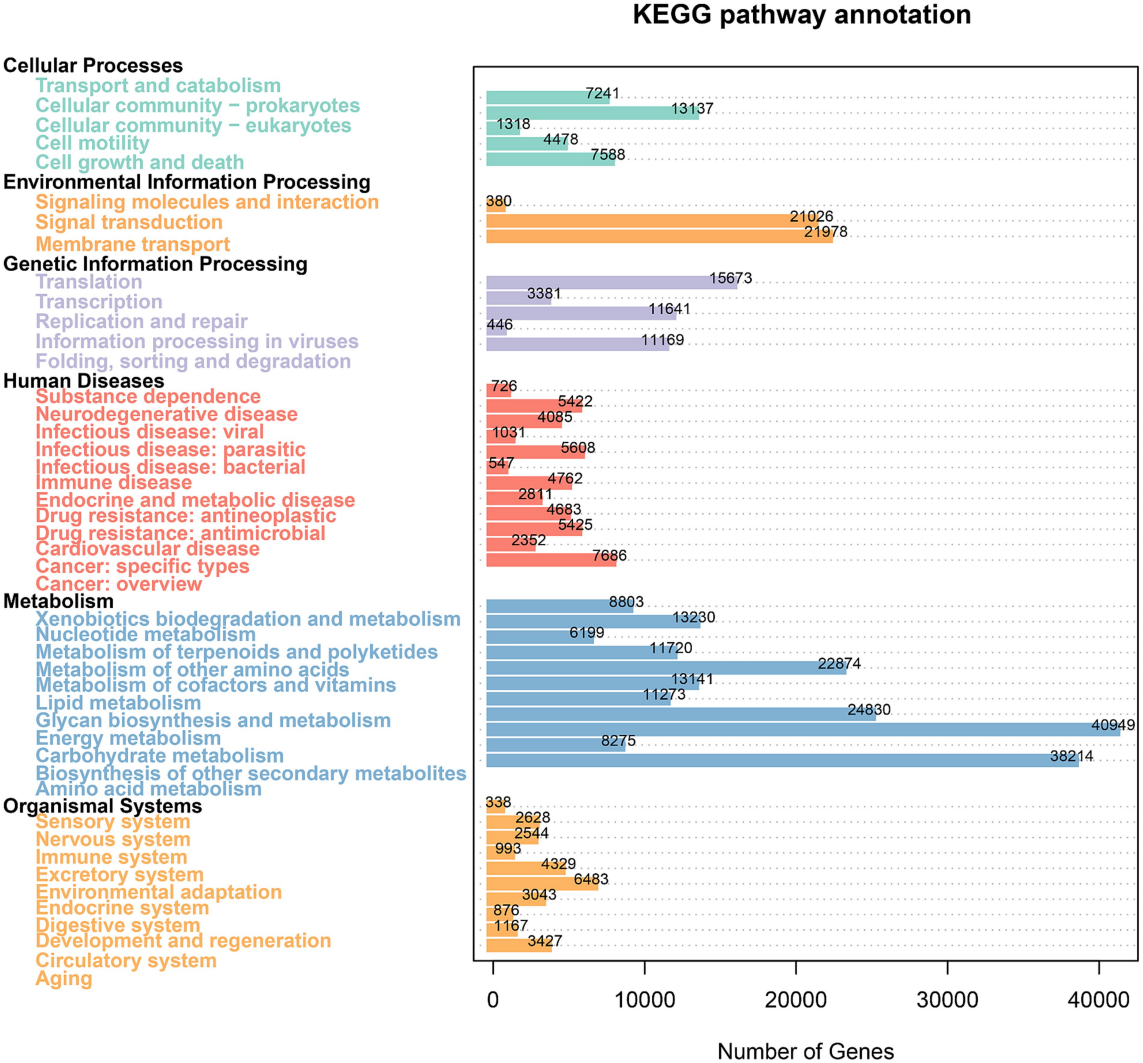
Analysis of groups contributions to KEGG.

**Figure 5:**
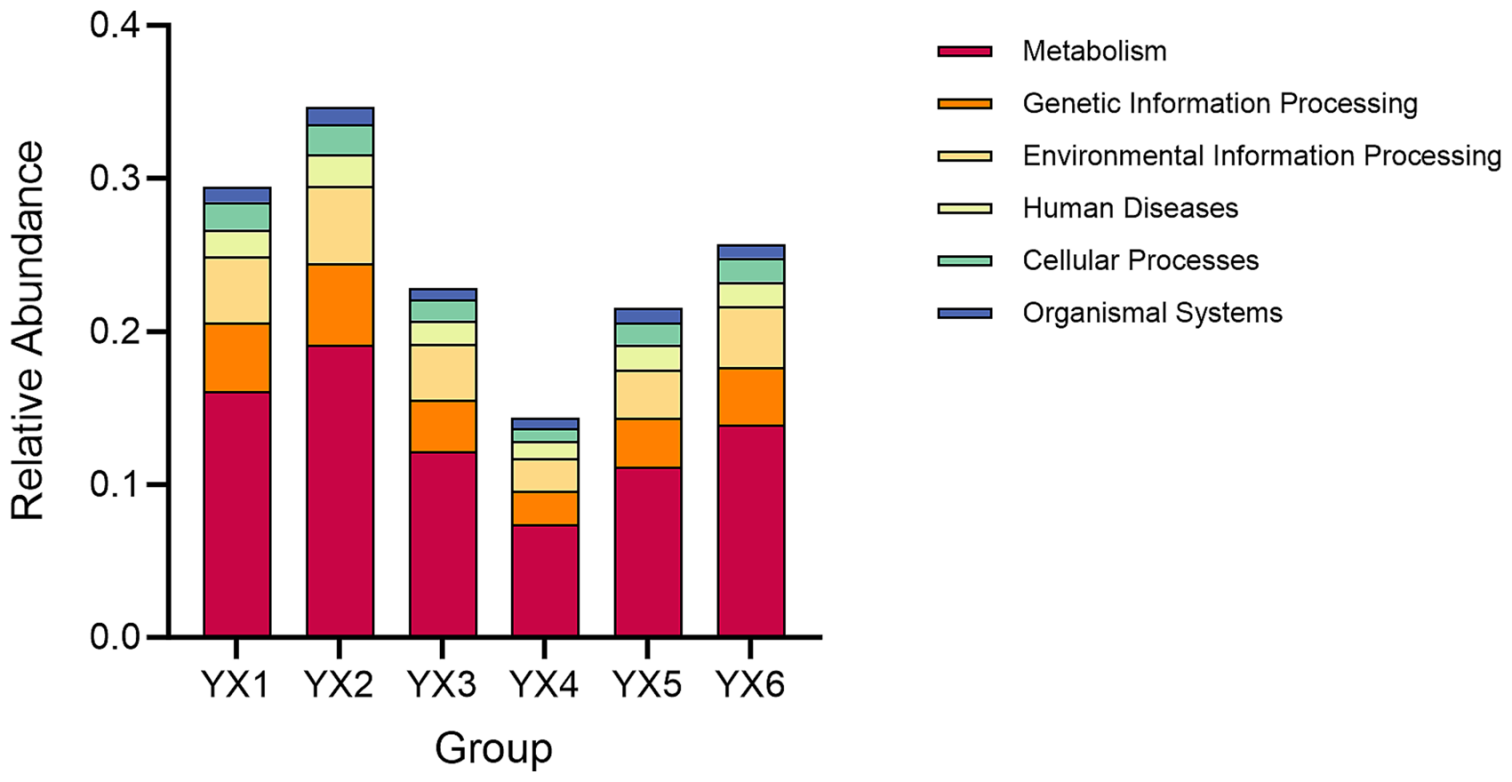
The number of genes related to KEGG metabolic pathways.

The KEGG database integrates gene and genome information with higher-level functional data, allowing for systematic analysis of gene function (Aoki-Kinoshita and Kanehisa, 2007). Cigar processing shapes microbial communities, impacting metabolic pathways and compound distributions. These metabolic dynamics are critical in shaping the chemical profiles that define a cigar’s flavor and quality. As shown in Figure 4, metabolism constituted a significant portion of functional activity in all groups, driving most of the between-group variability. Additionally, Figure 5 shows that the top five metabolic pathways in terms of relative abundance were carbohydrate metabolism, amino acid metabolism, energy metabolism, metabolism of cofactors and vitamins, and membrane transport. Furthermore, as shown in Supplementary Figure S1 “Unigenes KEGG” data, all the samples exhibited an enrichment of enzymes involved in the metabolism of terpenoids and polyketides, as well as related metabolic pathways. Consequently, differences in microbial composition across cigars intended for diverse purposes may lead to functional gene divergences, ultimately affecting flavor profiles.

**Figure 6:**
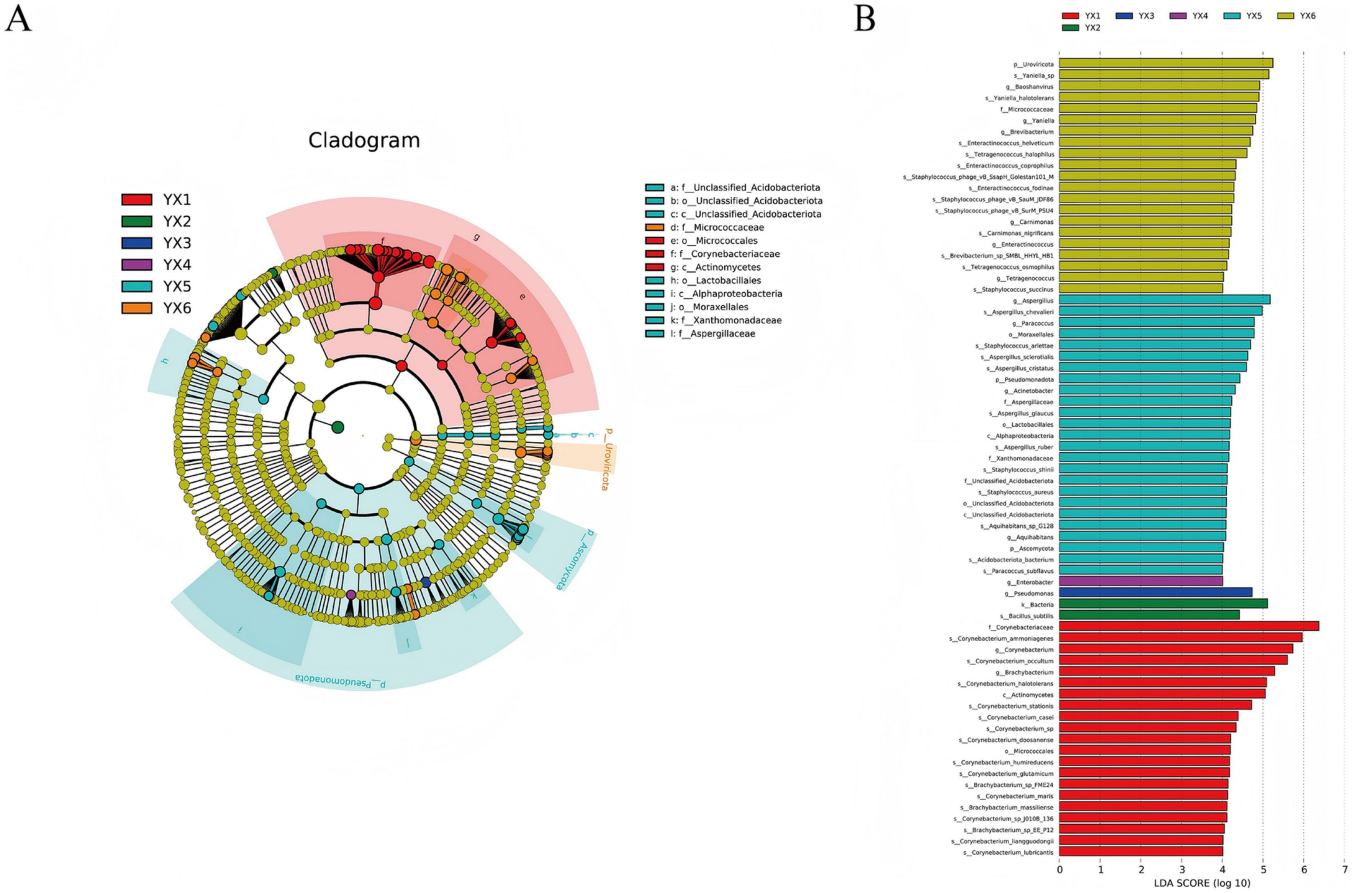
Differential microbes in different cigars. LEfSe **(A)** and LDA **(B)** analysis.

LEfSe analysis was conducted to identify significant differences below the phylum level (Figure 6A). Circles from inner to outer represent microbial classifications from phylum to genus, and each color denotes taxa that differ significantly among groups. Figure 6B demonstrates the Linear Discriminant Analysis (LDA) of microbial taxa across experimental groups (YX1–YX6), where higher LDA scores indicate stronger discriminatory power and taxonomic significance of specific microbes within their respective groups. From the LEfSe, we identified 3 classes, 4 orders, and 5 families showing notable divergence. For example, *Corynebacteriaceae* was enriched in YX1, *Lactobacillales* in YX5, and *Aspergillaceae* in YX6. Compared with other groups, YX5 displayed more pronounced microbial differences, and YX1 also showed unique microbiotas. The low LDA scores of the characteristic bacteria in groups YX2, YX3, and YX4 indicate a high microbial diversity in these groups, but relatively fewer specific contributing bacteria. Although the main characteristic bacteria of these groups are not as significant as YX1, YX5, and YX6, they may represent the transitional stage of microbial communities during the fermentation process. Such significant variations may be tied to differences in fermentation processes, community structure, and species balance, all of which impact cigar quality.

### 3.3 Profiles of volatile flavor compounds

We selected CAR/PDMS fiber for its suitability in capturing medium to low polarity compounds, optimizing target compound capture while minimizing interference. Mixed-coating fibers, like PDMS, enhance VOC coverage in multi-component analysis. The fiber’s excellent thermal stability ensures consistent GC–MS performance under high temperatures and supports repeated use. A DB-5 column, a non-polar option with a wide temperature range, was chosen to align with the fiber’s polarity, enabling efficient separation of medium to low polarity compounds with varying boiling points. This combination ensures reliable sensitivity and separation for the experiment.

To assess the differences in volatile flavor compounds among various cigars, HS-SPME-GC–MS analysis was conducted. Initially, 121 volatile organic compounds (VOCs) with identity scores exceeding 80 were preliminarily screened (Supplementary Table S2). These included 2 aldehydes, 8 ketones, 4 esters, 10 alkanes, 56 terpenes, 7 alkaloids, 9 alcohols, 1 phenol, and 24 other volatile components, with terpenes representing 46.28% of the VOCs.

**Figure 7:**
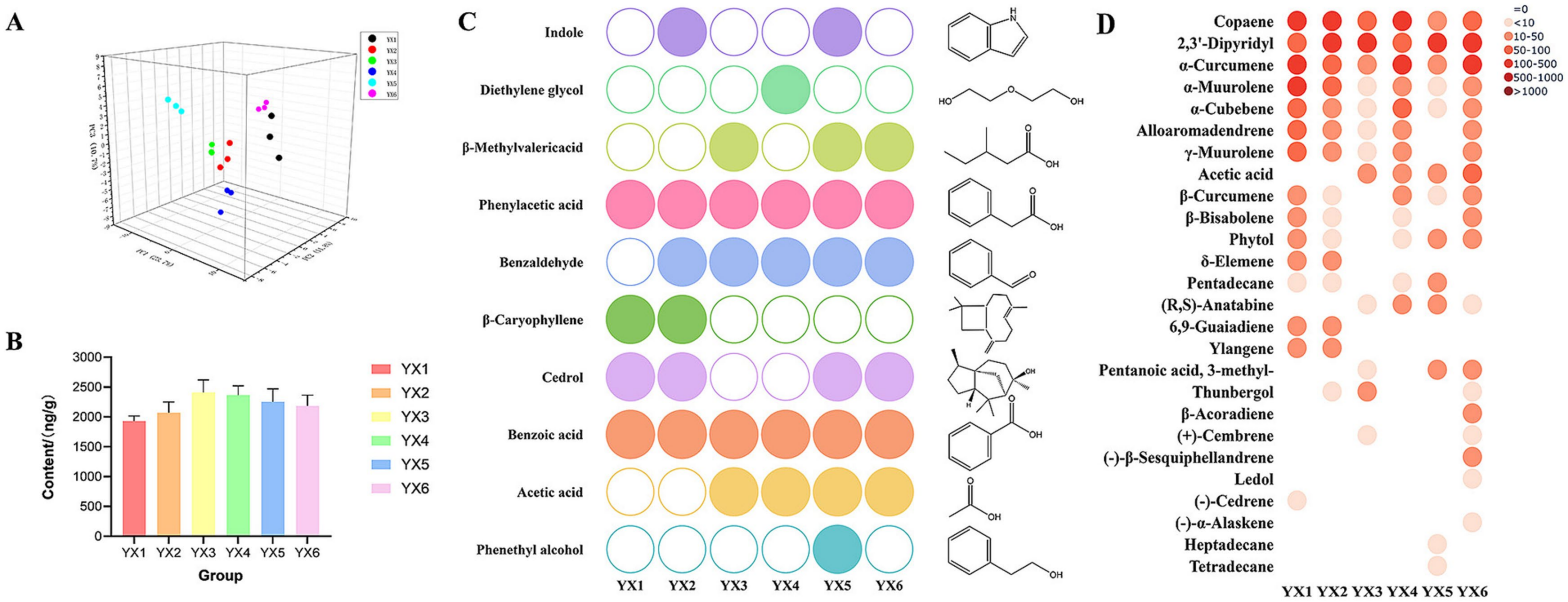
**(A)** PCA plot of six types of cigars based on aroma compounds; **(B)** content of nicotine in different group; **(C)** top 10 volatile compounds were identified using PCA; **(D)** FDR correction in VOC analysis.

Principal component analysis (PCA, Figure 7A) and heatmap (Supplementary Figure S1) were employed to examine distribution and diversity. PCA revealed close clustering of sample data points, suggesting high intra-group similarity. The heatmap showed that YX1, YX2, and YX6 exhibited more diverse VOC profiles: YX1 contained 68 distinct compounds, YX2 had 65, and YX6 had 66. In contrast, YX3 showed only 39 compounds, indicating a simpler volatile profile.

Following the application of PCA for dimensionality reduction of the experimental data, the cumulative variance contribution rate of the first three principal components reached 57.8%. Nicotine, phenethyl alcohol, and acetic acid emerged as the primary characteristic variables, with corresponding variance explanation rates of 29.6, 14.8, and 13.4%, respectively. This analysis indicates that these three characteristic compounds make significant contributions to the explanation of variability within the dataset. Nicotine emerged as the most abundant compound, with average relative contents ranging from 1783.47 to 2246.41 ng/g (Figure 7B), accounting for 29.72–55.00% of the total volatile organic compounds (TVOCs). In comparison, YX3 has the highest nicotine content, while YX1 has the lowest. After that, we selected the top 10 compounds with ratings other than nicotine. These metabolites are phenethyl alcohol, acetic acid, benzoic acid, cedrol, *β*-caryophyllene, benzaldehyde, phenylacetic acid, β-methylvalericacid, diethylene glycol, indole. The identified compounds have been found to significantly contribute to the overall aroma profile of tobacco leaves. Figure 7C presents the distribution of these 10 metabolites across different groups of cigars. Within this figure, distinctively colored circular symbols represent the 10 types of differential metabolites, whereas unfilled circles indicate the absence of detectable volatile metabolites in the respective samples. It is readily apparent that Cuban cigars YX5 and YX6 exhibit a richer profile of volatile components, which significantly contribute to their distinct aroma and sensory attributes.

The Kruskal-Wallis non-parametric rank sum test was used for multiple group comparisons, and the Benjamini-Hochberg (FDR) method was applied to control the type I error rate. A significance threshold of *α* = 0.05 was set, and a corrected *p*-value less than 0.05 was considered statistically significant. Ultimately, 26 differentiating compounds were screened out from 121 compounds (Figure 7D). Notably, all smaples have copaene, 2,3′-dipyridyl, α-curcumene, α- murolene and α-cubebene. Subsequently, the Wilcoxon rank-sum test was conducted, and the Bonferroni method was used for multiple-comparison correction (Table 2 Wilcoxon rank sum test results). The results indicated that for the five pairs, namely YX1 vs. YX3, YX1 vs. YX5, YX2 vs. YX3, YX3 vs. YX6, and YX5 vs. YX6, the *p*-values were less than 0.05, suggesting significant differences among them. In contrast, for pairs such as YX1 vs. YX2, YX1 vs. YX4, YX1 vs. YX6, YX2 vs. YX4, YX2 vs. YX6, YX3 vs. YX4, YX3 vs. YX5, YX4 vs. YX5, and YX4 vs. YX6, the p-values were greater than 0.05, indicating no significant differences.

**Table 2:** Wilcoxon rank sum test results.

Group	YX1	YX2	YX3	YX4	YX5
YX2	1.00000	–	–	–	–
YX3	4.1e-05	0.00205	–	–	–
YX4	0.17042	1.00000	0.50581	–	–
YX5	0.00438	0.13002	1.00000	1.00000	–
YX6	1.00000	1.00000	0.00016	1.00000	0.03150

### 3.4 Relationship between microbial communities and VOCs

Spearman correlation heatmaps and clustering analyses were conducted to investigate the interplay between microbial taxa and volatile flavor compounds. We focused on the top 10 most abundant microbial genera in each sample (YX1–YX6), examining their correlations with identified compounds (R > 0.05, *p* < 0.05). The resulting heatmaps and network diagrams (Supplementary Figures S3–S8) indicate that YX3 (Yunnan) and YX5 (Cuban) displayed relatively sparse correlations, suggesting potentially fewer direct links between microbes and VOCs in these two samples. By contrast, YX1 (Yunnan) and YX6 (Cuban) exhibited dense correlation networks, pointing to more intimate relationships. Interestingly, certain microbes seem played contrasting roles in different systems. For instance, *Baoshanvirus* and *Enterobacter* were positively correlated with VOCs in YX3 but showed negative correlations in the other five cigar types. *Aspergillus*, the sole fungal genus among the top 10 microbial genera, exhibited close associations with compounds across all six samples. Notably, in sample YX3, *Aspergillus* demonstrated significant positive correlations with the majority of compounds, whereas in the other five samples, its associations with compounds were predominantly characterized by negative correlations.

**Figure 8:**
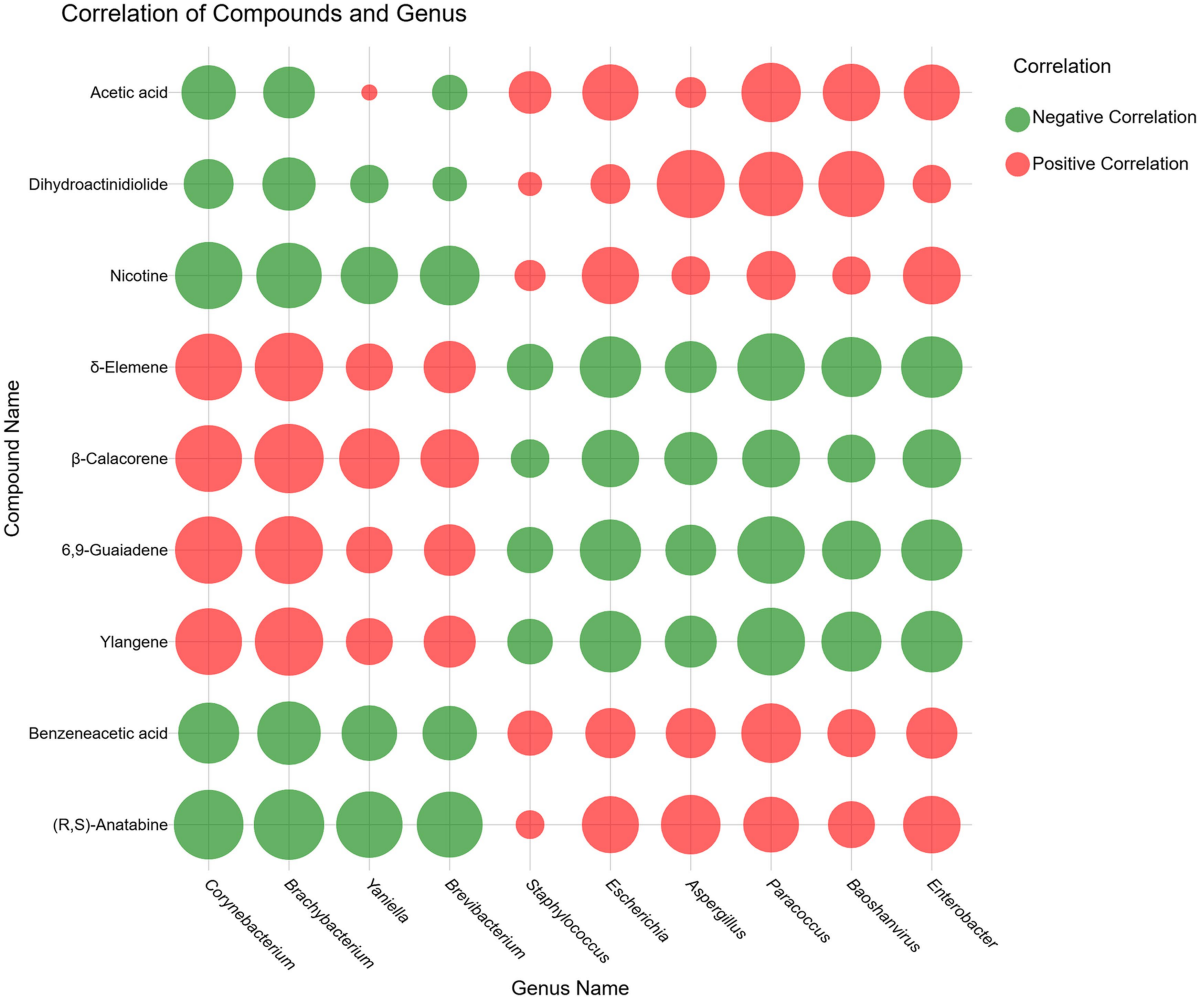
Microbial-VOCs correlation coefficient heatmap.

Meanwhile, we selected the top 9 compounds based on the absolute correlation values between each bacterial genus and the associated compounds and a heatmap (Figure 8) was constructed. As is evident from the graph, *Corynebacterium* and *Brachybacterium* exhibited significant positive correlations to the nine specified organic compounds, whereas *Staphylococcus* demonstrated weak correlations across all tested metabolites. Besides, the compounds *δ*-elemene, *β*-calacorene, 6,9-guaiadene, and ylangene exhibited an influence on the microorganism that was inversely related to the effects observed for acetic acid, dihydroactinidiolide, nicotine, benzeneacetic acid, and (R, S)-anatabine. Specifically, δ-elemene, β-calacorene, 6,9-guaiadene, and ylangene exhibited positive correlations with the four microbial genera, namely *Corynebacterium*, *Brachybacterium*, *Yaniella*, and *Brevibacterium*. In contrast, negative correlations were observed between these compounds and *Staphylococcus*, *Escherichia*, *Aspergillus*, *Baoshanvirus*, and *Enterobacter*. Conversely, (R, S)-anatabine, benzeneacetic acid, nicotine, and dihydroactinidiolide showed negative correlations with *Corynebacterium*, *Brachybacterium*, *Yaniella*, and *Brevibacterium*, while positive correlations were detected with *Staphylococcus*, *Escherichia*, *Aspergillus*, *Baoshanvirus*, and *Enterobacter*. Furthermore, acetic acid, while exhibiting a weak positive correlation specifically with *Yaniella*, generally displayed a correlation pattern consistent with that observed for the latter group of compounds.

## 4 Discussion

In this study, we investigated six cigar samples—four from Yunnan and two from Cuba—to elucidate the interplay between microbial community structure and aroma composition. Our findings highlight the critical role of microbial composition and geographic factors in shaping the aroma profiles of cigars, providing new insights into the microbial and metabolic mechanisms underlying flavor development. The dominance of *Bacillus* spp. across all samples underscores its pivotal role in cigar fermentation, aligning with previous studies, which have demonstrated that *Bacillus* not only represents the dominant microbial genus in cigars but also possesses enzymatic capabilities to transform pungent precursors into desirable aromatic compounds (English et al., 1967; Xueru et al., 2022; Brandsch, 2006). Compared to other major cigar-producing regions, the microbial communities in cigars exhibit significant regional differences. For instance, in Yunnan cigars, Staphylococcus constitutes approximately 20% of the total microbial genera, whereas in Brazilian cigars, this proportion reaches as high as 79.66%. Additionally, the abundance of Aspergillus in Yunnan cigars is markedly lower than the 78.31% observed in Indonesian cigars. These disparities likely reflect the unique climatic conditions, fermentation processes, and tobacco raw materials characteristic of each region, which profoundly influence the composition and distribution of microbial communities (Zheng et al., 2022).

However, our analysis also revealed region-specific microbial signatures. *Brachybacterium* was identified as a characteristic microorganism in YX1 and YX2, and microbial taxa affiliated with the family *Micrococcaceae* predominated in Yunnan YX1, while the microbial communities in YX3 and YX4 were comparatively less diverse. Notably, YX4 exhibited a relatively high abundance of *Aspergillus*. Additionally, *Paracoccus* was identified as a characteristic microorganism in YX5, whereas *Baoshanvirus* was unique to YX6. *Brachybacterium* has ability to produce polyphenol oxidase, which facilitates the formation of pigments such as theaflavins and various aromatic compounds (Pan et al., 2023; Zuo et al., 2024). *Micrococcaceae* functionally linked to proteolytic and amino acid metabolic pathways, may underpin the formation of the initial flavor profile in cigar fermentation (Sims et al., 1986). Besides, the lower microbial abundance observed in YX3 and YX4 suggests a more advanced stage of fermentation, potentially reflecting their progression into a final equilibrium phase. The relatively high abundance of *Aspergillus* in YX4 may be attributed to the higher humidity levels in its fermentation environment. Moreover, the presence of *Paracoccus* is capable of effectively degrading phthalate compounds, playing a role in the removal of organic pollutants generated during the cigar production process (Xu et al., 2020). This suggests that the cultivation environment of YX5 may be contaminated to some extent. Currently, there are no reports demonstrating the function of *Baoshanvirus* in tobacco, it may represent a local microbial adaptation specific to the Vuelta Abajo region. These findings suggest that regional factors such as climate, soil, and fermentation practices significantly influence microbial diversity.

In terms of VOCs, nicotine plays a central role in cigar quality (Zhang et al., 2025b). Beyond its irritant moisturizing effect, is the most abundant compound across all samples, and it is also a key marker of product consistency (Yi et al., 2024). Nicotine biosynthesis involves the formation of a pyridine ring and a pyrrolidine ring. Key enzymes for the pyridine ring—aspartate oxidase (AO, EC 1.4.3.16), quinolinate synthase (QS, EC 2.5.1.72), and quinolinate phosphoribosyl transferase (QPT, EC 2.4.2.19)—were abundantly detected in sample YX5 and associated with *Paracoccus*, *Staphylococcus*, and *Enterobacter* (Brandsch, 2006). For the pyrrolidine ring, ornithine decarboxylase (ODC, EC 4.1.1.17) was found in *Enterobacter*, *Aspergillus*, *Escherichia*, and *Paracoccus*; PMT (EC 2.1.1.53) was absent in YX1 and YX2; and N-methylputrescine oxidase (MPO, EC 1.4.3.-) was abundant in YX5 and present at lower levels in YX6. These results suggest a potential positive correlation between nicotine levels and the presence of *Paracoccus*, *Staphylococcus*, *Enterobacter*, *Aspergillus*, and *Escherichia*. Additionally, in the nicotine degradation pathway, MPO1 (EC 1.4.3.21)—detected in all samples and linked to *Brevibacterium*—indicates that *Brevibacterium* may contributes to nicotine breakdown, aligning with its negative correlation with nicotine concentration.

Ylangene, a sesquiterpene with distinct floral and fruity notes, contributes significantly to cigar aroma complexity (Huayi et al., 2024). In this study, ylangene exhibited positive correlations with *Brachybacterium* and *Corynebacterium*, but negative correlations with *Aspergillus*, *Enterobacter*, *Escherichia*, *Paracoccus*, and *Baoshanvirus*. The key biosynthetic enzyme, sesquiterpene synthase (TPS2, EC: 4.2.3.-), was identified in YX3, YX4, YX5, and YX6. However, HS-SPME-GC–MS analysis revealed that ylangene was only detected in YX1 and YX2, not in the TPS2-positive samples. This discrepancy suggests that TPS2 expression alone may not determine ylangene presence, possibly due to regulatory or metabolic constraints. Although *Aspergillus* is a dominant fungus in tobacco fermentation, its weak correlation with ylangene implies a limited role in its biosynthesis. Nonetheless, *Aspergillus* contributes to overall aroma formation by producing enzymes such as amylase, protease, and cellulase, which degrade macromolecules in tobacco leaves into precursors of small aromatic compounds (Zhao et al., 2024).

(R, S)-Anatabine, a minor pyridine alkaloid in tobacco, is primarily synthesized via enzymatic pathways similar to those of nicotine. In this process, lysine is first converted to cadaverine by lysine decarboxylase (LDC), which is then cyclized by cyclase enzymes to form a piperidine ring structure (Leete, 1975). Degradation of (R, S)-anatabine has been demonstrated in *Arthrobacter*, which contains nicotine dehydrogenase, and in *Pseudomonas*, which harbors NicA2 (EC: 1.4.2.2) homologous enzymes. These enzymes have also been detected in YX2 and YX5 (Ruan et al., 2005; Sun et al., 2015). Experimental analyses reveal that the top 10 bacterial strains exhibit similar degradation patterns for (R, S)-anatabine and nicotine. Notably, *Yaniella*, *Brevibacterium, Corynebacterium*, *Brachybacterium*, and show negative correlations with both compounds, suggesting they likely possess NicA2 homologs and nicotine dehydrogenase, enabling the breakdown of tobacco alkaloids.

The KEGG pathway analysis further elucidated the metabolic basis of these microbial-chemical interactions. Pathways related to carbohydrate metabolism, amino acid biosynthesis, and lipid degradation were enriched in the microbial communities, producing precursors such as phenylalanine and tyrosine, which are critical for the synthesis of benzeneacetic acid and other aromatic compounds. Benzeneacetic acid is an intermediate in the degradation of phenylalanine, in which phenylalanine is converted to benzeneacetic acid through a series of reactions. In some bacteria, such as *Escherichia coli*, phenylacetate can be further metabolized into other compounds. The process involves the conversion of benzeneacetic acid to phenylacetyl-CoA (Ju et al., 2021). In addition, the phenylalanine metabolic pathway can also produce other aromatic compounds, such as benzoic acid and coumarin (Moore et al., 2002). Carbohydrate metabolism intermediates serve as substrates for various biosynthetic processes, including those producing aromatic amino acids (Pittard et al., 2005; Zhao et al., 2013).

Indole is a compound with a dual odor profile: it smells fecal at high concentrations but floral when highly diluted (Zeng et al., 2016). Its biosynthesis mainly derives from tryptophan metabolism. Typically, tryptophan is deaminated by tryptophanase to form indole-3-pyruvate, which is decarboxylated to indole-3-acetaldehyde and then oxidized to indole. Alternatively, tryptophan can be converted to indole-3-acrylic acid and subsequently decarboxylated to produce indole (Tang et al., 2023). This pathway varies across organisms: *Escherichia coli* synthesizes indole via tryptophanase (TnaA, EC: 4.1.99.1) (Watanabe and Snell, 1972); *Bacillus subtilis* uses tryptophan deaminase to generate indole-3-acrylic acid (Hoch and Crawford, 1973); *Pseudomonas aeruginosa* produces indole from tryptophan in soil and aquatic environments (Jayaraman et al., 2023); while *Aspergillus niger* converts anthranilic acid into indole through a distinct route (Arora et al., 2015). These findings illustrate the metabolic diversity underlying indole biosynthesis across taxa.

Acetic acid is a ubiquitous organic acid synthesized by various microorganisms through distinct enzymatic pathways. In aerobic conditions, *Acetobacter* and *Gluconobacter* oxidize ethanol to acetic acid via alcohol dehydrogenase (ADH, EC: 1.1.1.1) and aldehyde dehydrogenase (ALDH, EC: 1.2.1.3) (Mamlouk and Gullo, 2013; Mientus et al., 2017). Anaerobic microbes such as methanogens and sulfate-reducing bacteria also generate acetic acid through the acetyl-CoA pathway (Ragsdale, 2008). Metagenomic analysis revealed the presence of ADH and ALDH genes in *Enterobacter*, *Paracoccus*, *Aspergillus*, *Staphylococcus*, and *Yaniella*. Among them, *Enterobacter* was enriched in YX3 and YX5, *Aspergillus* was found in all samples, and *Staphylococcus* appeared only in YX5. In contrast, YX1 and YX2 showed lower abundance of these enzyme-producing genera. The acetate-degrading enzyme, acetyl-CoA synthetase (EC: 6.2.1.1), was annotated in *Corynebacterium*, *Brachybacterium*, and *Brevibacterium*, consistent with their negative correlation with acetate levels.

Globally, Cuban cigars are renowned for their craftsmanship, premium tobacco, and intricate fermentation process (Viola et al., 2016). In recent years, however, the global cigar landscape has diversified, with Nicaragua, the Dominican Republic, and Honduras gaining recognition through regional strengths and process innovation. Yunnan cigars from China have also emerged as strong competitors, driven by advances in fermentation science, improved tobacco cultivation, and policy support (Shuoli et al., 2023). Comparative analysis of four Yunnan and two Cuban cigars revealed that certain Yunnan samples exhibited microbial abundances comparable to or exceeding those of Cuban cigars, likely due to Yunnan’s distinctive climate, mineral-rich and soil (Wu et al., 2024). Moreover, some Yunnan cigars demonstrated notable aroma diversity and higher VOC concentrations, indicating potential quality attributes comparable to Cuban cigars (Yang et al., 2022). Overall, cigar flavor and quality result from a complex interplay of factors including terroir, tobacco variety, microbial ecology, and fermentation practices.

In summary, this study provides preliminary insights into the microbial communities and VOC profiles associated with Yunnan cigars. These findings may contribute to a deeper understanding of how microbial and chemical diversity might influence cigar quality. As future research continues to investigate the complex interactions between microbial metabolism and aroma formation, additional factors that shape cigar characteristics may be uncovered. Notably, the observed microbial diversity and VOC richness in Yunnan cigar samples appear to be comparable to those reported in Cuban cigars, which might suggest that regional cigar profiles are influenced by more than just geographic origin. These results may offer a new perspective for exploring the microbial basis of product quality and could inform future strategies in cigar fermentation and development.

## Data Availability

The original contributions presented in the study are publicly available. This data can be found here: accession number: PRJNA 1261466.

## Footnotes

^1^ https://github.com/OpenGene/fastp

^2^ http://topaz.gatech.edu/GeneMark/

^3^ http://www.bioinformatics.org/cd-hit/

^4^ https://www.nist.gov
