# Supplementary material for: Unraveling the microbiome–aroma Nexus: a metagenomic and volatile compound analysis of Yunnan cigars
Source: Front Microbiol. 2025 Jul 9;16:1597501. doi: 10.3389/fmicb.2025.1597501 (PMC12283659; doi:10.3389/fmicb.2025.1597501)
Supplement: Supplementary file 1 [file Table_1.docx]

Supplementary Material

# Supplementary Figures and Tables

**Table S1 The 121 volatile compounds**

| **NO.** | **CAS** | **Description** | **Compounds** |
| --- | --- | --- | --- |
| 1 | 054274-73-6 |  | Nephthene |
| 2 | 029419-55-4 |  | (1's,2's)-Nicotine-N'-oxide |
| 3 | 036577-33-0 | Special aromatic odor | 6,9-Guaiadiene |
| 4 | 729602-94-2 |  | β-Acoradiene |
| 5 | 099529-78-9 |  | β-Alaskene |
| 6 | 028400-12-6 |  | (-)-α-Alaskene |
| 7 | 267665-20-3 |  | Cadinα-3,5-diene |
| 8 | 123123-37-5 | Slightly sweet with a salty taste | α-Salinen |
| 9 | 038230-60-3 | Warm, spicy, naturally cool, with a mint like aroma | Cubebol |
| 10 | 019903-73-2 | Woody fragrance | Cedran-8-ol |
| 11 | 023445-02-5 |  | (-)-Cubebol |
| 12 | 072203-99-7 |  | (+)-Axenol |
| 13 | 1000432-20-1 | Woody aroma | (E)-β-Farnesene |
| 14 | 018794-84-8 | Floral fragrance, apple fragrance | (E)-β-Famesene |
| 15 | 028976-67-2 | Special odor with a slightly bitter taste | β-Curcumene |
| 16 | 000481-34-5 | Pine wood aroma | α-Cadinol |
| 17 | 021391-99-1 | Woody aroma | α-Calacorene |
| 18 | 017699-14-8 | The fragrance of plants and trees | α-Cubebene |
| 19 | 003691-12-1 | Wood fragrance and herbal aroma | α-Guaiene |
| 20 | 010208-80-7 | Woody fragrance | α-Muurolene |
| 21 | 000495-61-4 | Warm woody, citrus, floral, fruity, green, and sweet creamy aromas | β-Bisabolene |
| 22 | 030021-74-0 | Herbal scent | γ-Muurolene |
| 23 | 005937-11-1 | woody | T-Cadinol |
| 24 | 019912-62-0 | Herbal spicy honey scent | T-Muurolol |
| 25 | 002743-90-0 |  | (R,S)-Anatabine |
| 26 | 022469-52-9 |  | (+)-Cyclosativene |
| 27 | 1000156-99-5 |  | 1,3,5-Cycloheptatriene, 2,5-diethyl-7,7-dimethyl- |
| 28 | 001898-13-1 | Odor of cypress and sandalwood | (+)-Cembrene |
| 29 | 015423-57-1 | A mixture of soil and spicy odor | Germacrene B |
| 30 | 016729-00-3 |  | Isocadinene |
| 31 | 016729-01-4 |  | D-Amorphene |
| 32 | 000451-55-8 |  | Curcumene <γ-> |
| 33 | 000469-61-4 | Pine needle odor | (-)-Cedrene |
| 34 | 001461-03-6 |  | Himachalene |
| 35 | 013744-15-5 | Aromatic, slightly spicy and bitter | Cubebene |
| 36 | 000489-40-7 | Woody aroma | (-)-α-Gurjunene |
| 37 | 021747-46-6 | Chrysanthemum wax floral fragrance | (+)-Ledene |
| 38 | 001128-54-7 |  | 3-Methyl-1-phenyl-1H-pyrazole |
| 39 | 020189-42-8 |  | 1H-Pyrrole-2,5-dione |
| 40 | 015356-74-8 | Musk | (±)-Dihydroactinidiolide |
| 41 | 017092-92-1 | Fruit and floral aromas | R-Dihydroactinidiolide |
| 42 | 000581-50-0 |  | 2,3'-Dipyridyl |
| 43 | 010236-16-5 | Wax | (E)-Phytyl acetate |
| 44 | 000502-69-2 | Floral | Hexahydrofarnesyl acetone |
| 45 | 015769-88-7 |  | Nicotone |
| 46 | 1000400-21-5 |  | 4,4,7-Trimethyl-2-chromanone |
| 47 | 1000245-55-1 | The aroma of tobacco, caramel, and roasted pineapple | Phytofuran |
| 48 | 013788-84-6 |  | 3-Methyl-4-phenylpyrazole |
| 49 | 050277-34-4 | Sweet woody spices and ding aroma | β-Calacorene |
| 50 | 073365-77-2 |  | Cadinenol |
| 51 | 000762-29-8 | Plant volatiles | Farnesalacetone |
| 52 | 001117-52-8 | Ether odor | E,E-Farnesylacetone |
| 53 | 000270-91-7 |  | 5H-1-Pyrindine |
| 54 | 019895-35-3 | Rich sweetness and floral fragrance | 2-Hepten-5-olide |
| 55 | 001604-28-0 | Citrus flavor, fruity flavor | 6-Methyl-3,5-heptadiene-2-one |
| 56 | 000103-82-2 | Spicy floral aroma | Acetic acid |
| 57 | 025246-27-9 | Woody aroma | Alloaromadendrene |
| 58 | 000489-39-4 | Prominent fruity aroma | Aromandendrene |
| 59 | 003691-11-0 | Pine wood aroma | α-Bulnesene |
| 60 | 000100-52-7 | Bitter almond flavor, fragrant when burning | Benzaldehyde |
| 61 | 000644-30-4 | The scent of herbs | α-Curcumene |
| 62 | 000122-78-1 | Green sweet floral hyacinth clover honey cocoa | Benzeneacetaldehyde |
| 63 | 000103-82-2 | Spicy floral aroma | Benzeneacetic acid |
| 64 | 000065-85-0 | Balsamic | Benzoic acid |
| 65 | 000140-29-4 | Bitter almond aroma | Benzyl nitrile |
| 66 | 000514-14-7 |  | Fenchene |
| 67 | 017699-05-7 | Lemon fragrance, Volatile components | Bergamotene |
| 68 | 054324-03-7 |  | Bicyclosesquiphellandrene |
| 69 | 000087-44-5 | Spicy, woody, citrus, camphor, and mild clove aromas | Caryophyllene |
| 70 | 019870-75-8 |  | Cedrane, 8-propoxy- |
| 71 | 000077-53-2 | Cedarwood woody dry sweet soft | Cedrol |
| 72 | 003856-25-5 | A fresh blend of pine, needle, and resin scent | Copaene |
| 73 | 029837-12-5 | Mango flavor | Cubenene |
| 74 | 000515-13-9 | Spicy | β-Elemene |
| 75 | 000639-99-6 | Wood fragrance and spicy aroma | Elemol |
| 76 | 020307-83-9 | Herbal, fruity, woody | (-)-β-Sesquiphellandrene |
| 77 | 020307-84-0 | Anise odor | δ-Elemene |
| 78 | 000629-94-7 | Wax flavor | Eicosane |
| 79 | 041702-63-0 |  | Epizonarene |
| 80 | 000111-46-6 | Acrid odour | Ethanol, 2,2'-oxybis- |
| 81 | 000350-03-8 | Floral fragrance, nutty aroma, similar to the aroma of popcorn, with a sweet taste | Ethanone, 1-(3-pyridinyl)- |
| 82 | 001122-54-9 |  | Ethanone, 1-(4-pyridinyl)- |
| 83 | 023986-74-5 | Aromatic odor | Germacrene D |
| 84 | 013287-23-5 |  | Heptadecane |
| 85 | 000638-36-8 |  | Hexadecane |
| 86 | 006753-98-6 | Wood, amber and musk fragrance | Humulene |
| 87 | 000120-72-9 | Burnt, mothball | Indole |
| 88 | 000577-27-5 | Orange, floral fragrance | Ledol |
| 89 | 038818-55-2 | Sweet aroma of tobacco | Megastigmatrienone |
| 90 | 039029-41-9 | Herbal woody fragrance | (+)-γ-Cadinene |
| 91 | 016728-99-7 | Fruity | Naphthalene |
| 92 | 000483-77-2 | Vanilla fragrance | Calamenene |
| 93 | 000483-76-1 | Herbal | δ-Amorphene |
| 94 | 000483-75-0 | Wood odor | (-)-α-Muurolene |
| 95 | 024406-05-1 | Wood odor | α-Cadinene |
| 96 | 017627-24-6 |  | （+）-α-Muurolene |
| 97 | 000483-78-3 |  | Cadalene |
| 98 | 017066-67-0 | Herbal | β-Selinene |
| 99 | 000504-96-1 | Clear aroma | Neophytadiene |
| 100 | 1000285-43-6 | Sweet and delicate orange blossom scent | Nerolidol |
| 101 | 1000285-43-6 |  | Nerolidol 2 |
| 102 | 000487-19-4 | Spicy taste | Nicotyrine |
| 103 | 000629-62-9 | Volatility | Pentadecane |
| 104 | 001921-70-6 |  | Pristane |
| 105 | 000105-43-1 | Stimulating acidic cheese odor | Pentanoic acid, 3-methyl- |
| 106 | 000060-12-8 | Has a rose fragrance | Phenylethyl Alcohol |
| 107 | 000150-86-7 | Aromatic odor | Phytol |
| 108 | 000054-11-5 | Stimulating ammonia odor with weak burnt bitterness | Nicotine |
| 109 | 000532-12-7 | Nutty, sweet | Myosmine |
| 110 | 001008-88-4 |  | Pyridine, 3-phenyl- |
| 111 | 000629-59-4 | Gentle wax flavor | Tetradecane |
| 112 | 025269-17-4 | Sulfur odor | Thunbergol |
| 113 | 000508-32-7 | The smell of camphor | Tricyclene |
| 114 | 014912-44-8 | Spice | Ylangene |
| 115 | 041929-05-9 | Spicy | Zonarene |
| 116 | 028973-97-9 | Wood fragrance, citrus, herbs, and sweetness | cis-β-Farnesene |
| 117 | 157477-72-0 |  | cis-Muurola-4(15),5-diene |
| 118 | 1000365-95-4 |  | cis-muurola-3,5-diene |
| 119 | 095910-36-4 | Aromaticity, coolness, soil and earthy taste | Isoledene |
| 120 | 013474-59-4 | Wooden, warm toned, tea flavored | (E)-α-Bergamotene |
| 121 | 073209-42-4 |  | trans-Calamenene |

Odour descriptions are based on The Good Scents Company (http://www.thegoodscentscompany.com/search2.html) and Flavor Ingredient Library (<https://www.femaflavor.org/flavor-library>

# Supplementary Figures


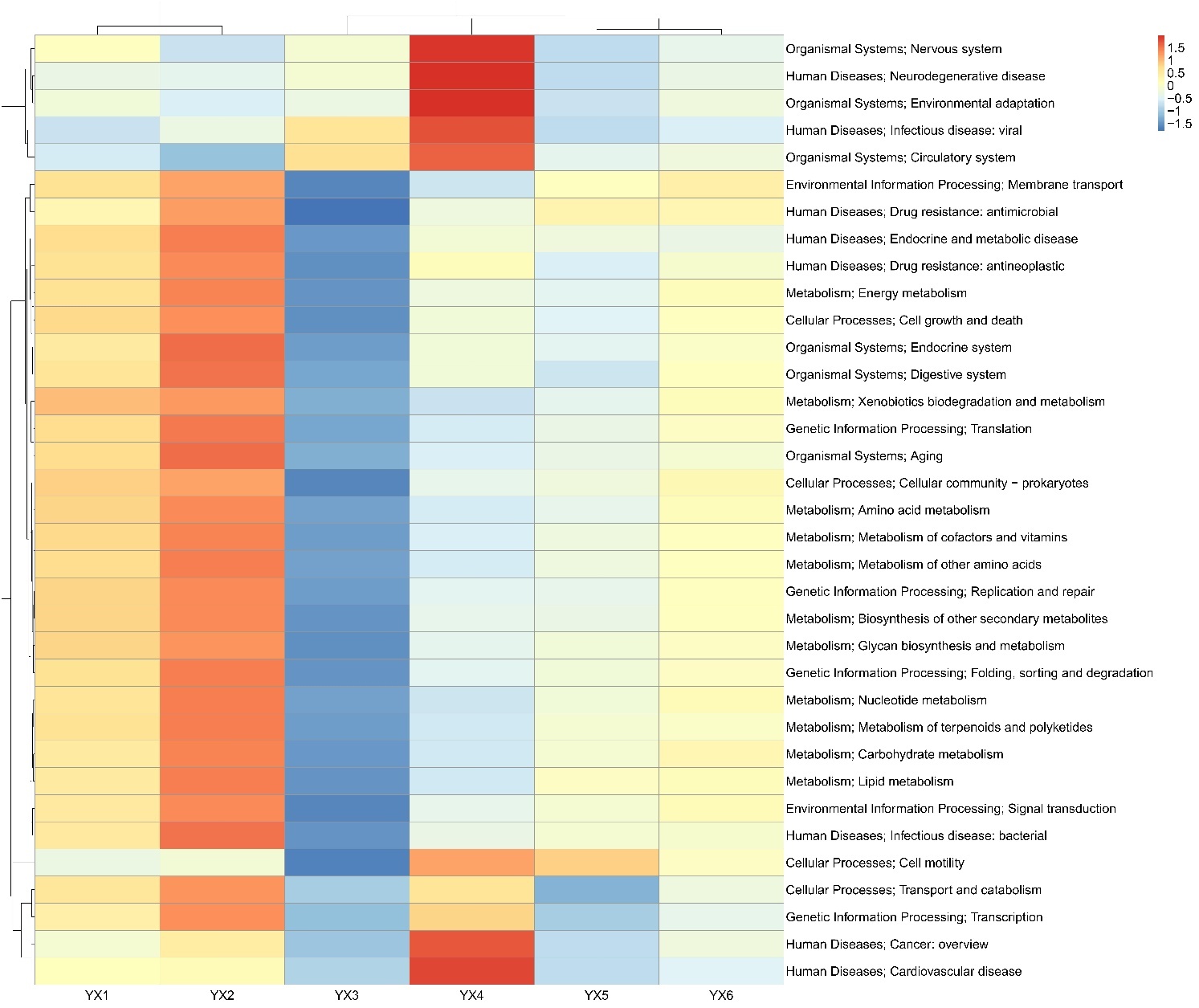


**Supplementary Figure 1.** Cluster analysis of relative functional abundance of KEGG.

##
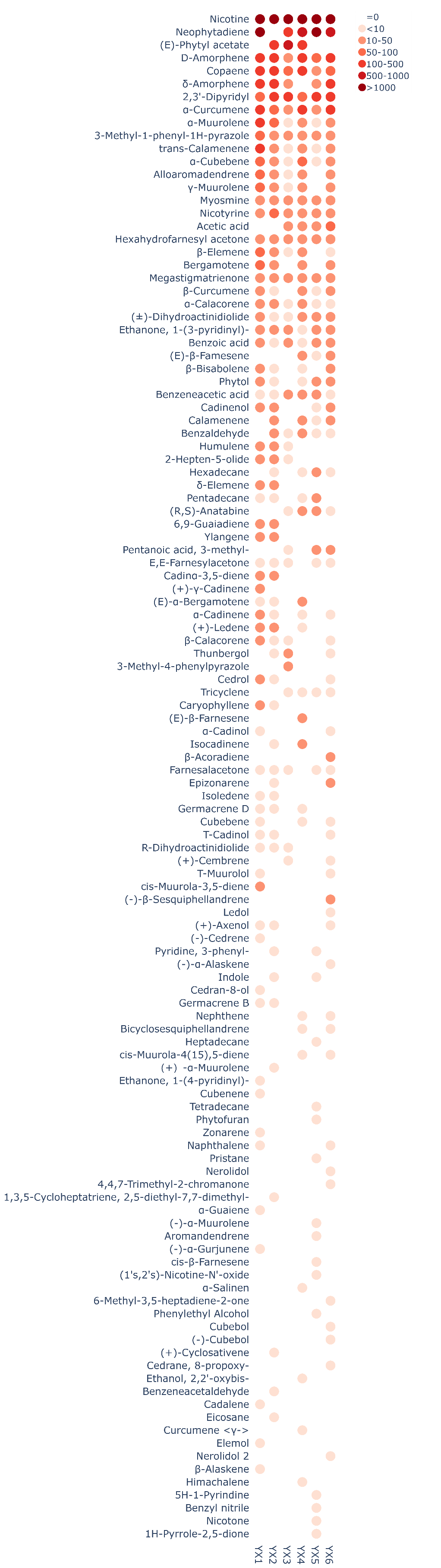


**Supplementary Figure 2.** Heatmap of 121 volatile compounds in six groups.


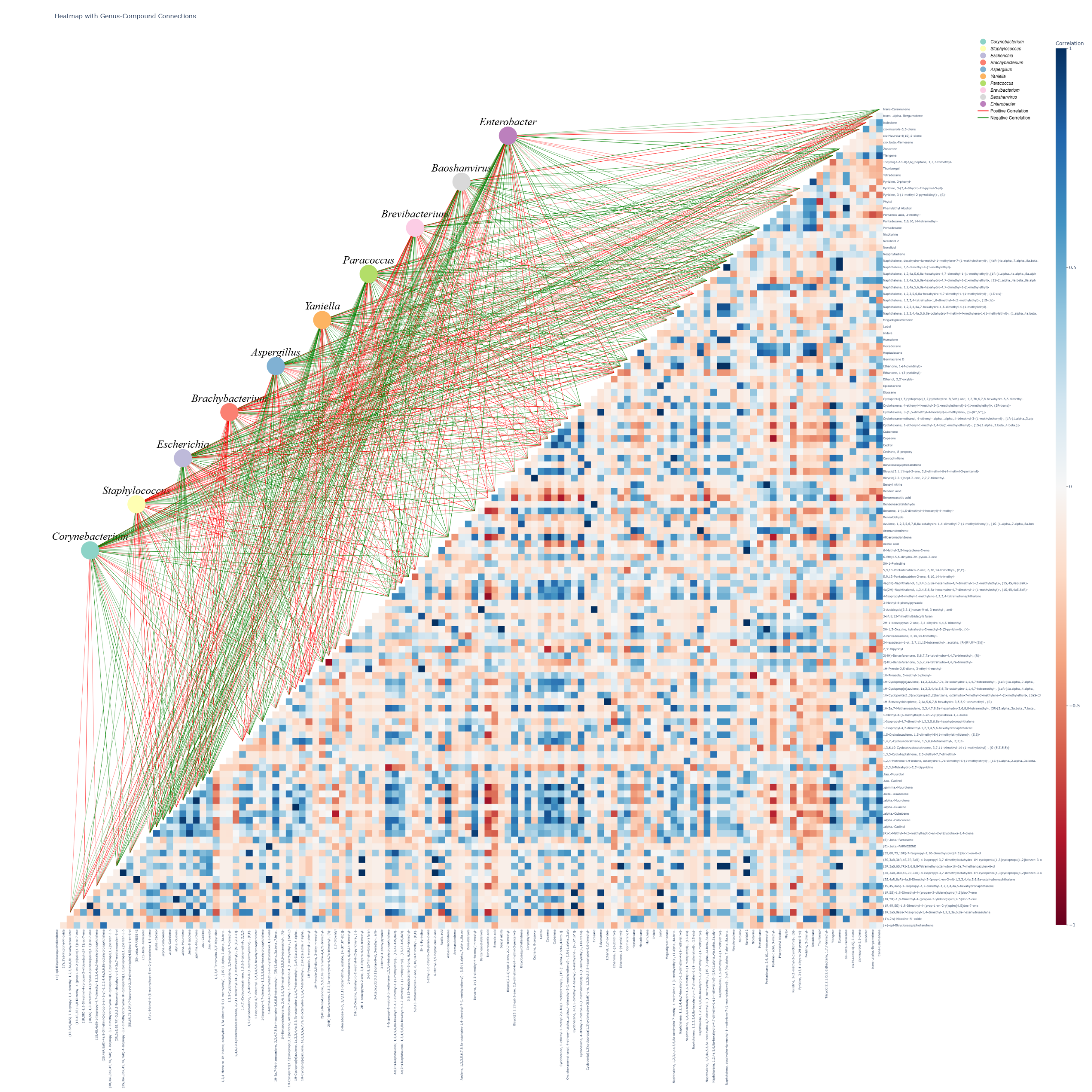


**Supplementary Figure 3.** The relationship between top 10 microorganism and VOCs was analyzed by heat map and network map with Spearman in YX1.


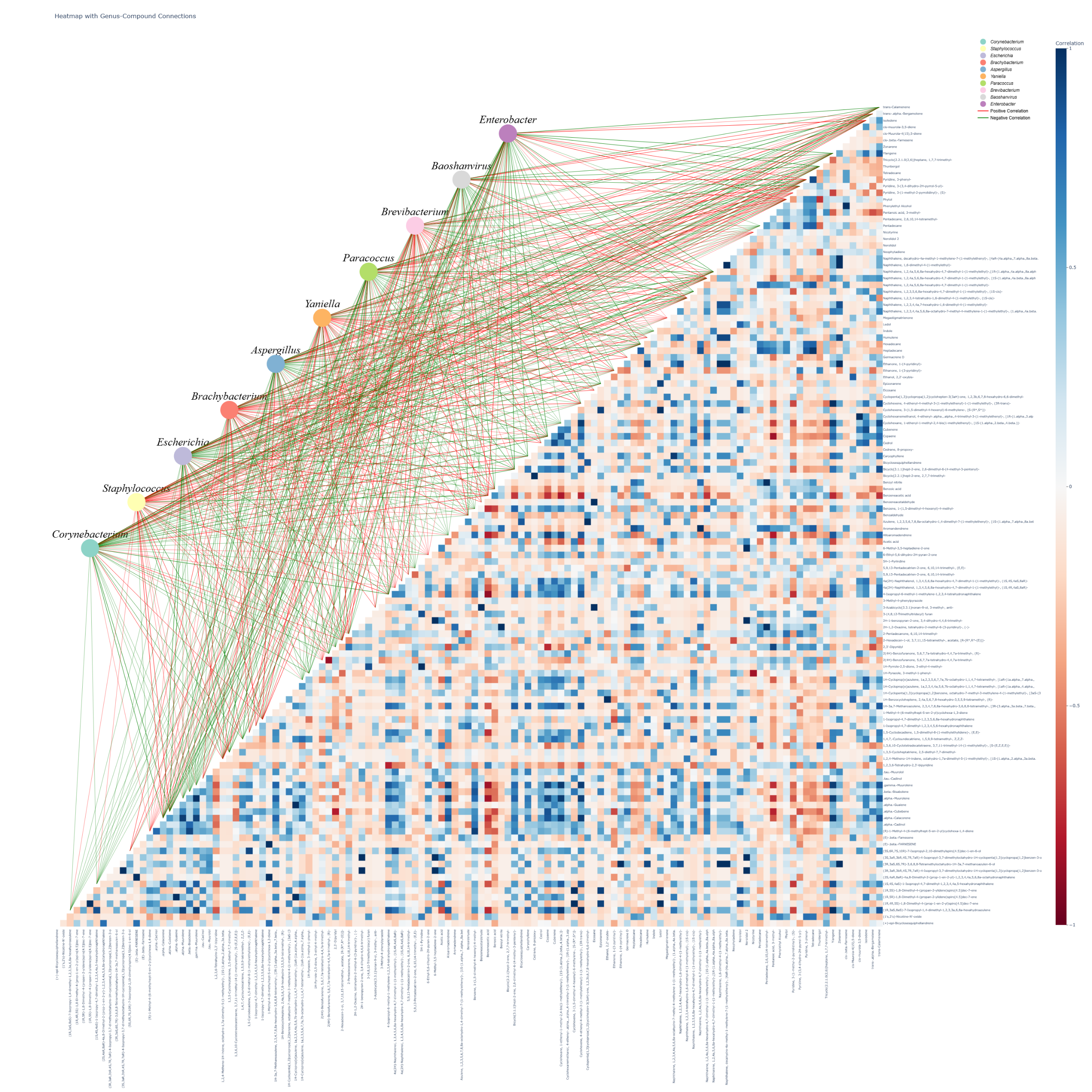


**Supplementary Figure 4.** The relationship between top 10 microorganism and VOCs was analyzed by heat map and network map with Spearman in YX2.


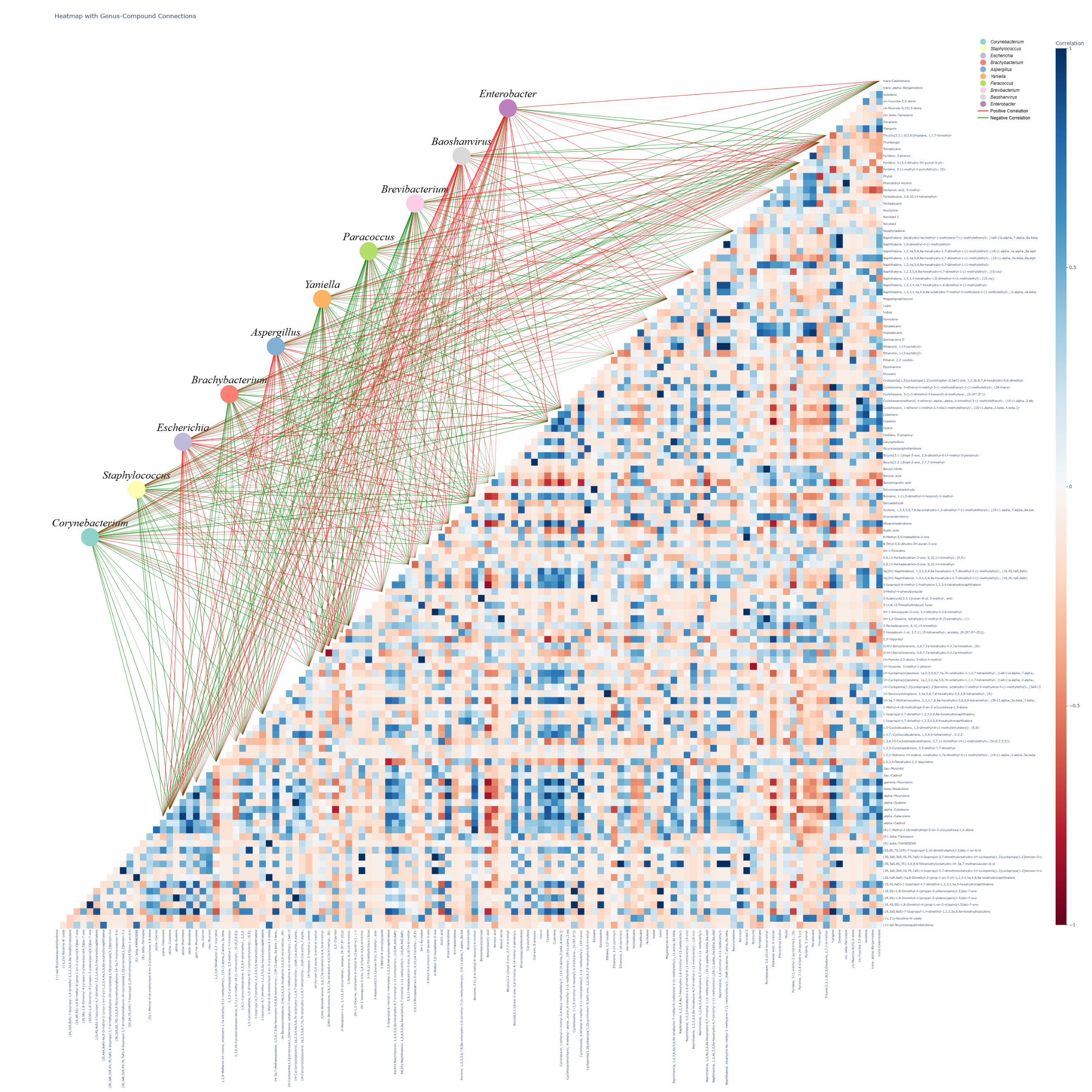


**Supplementary Figure 5.** The relationship between top 10 microorganism and VOCs was analyzed by heat map and network map with Spearman in YX3.


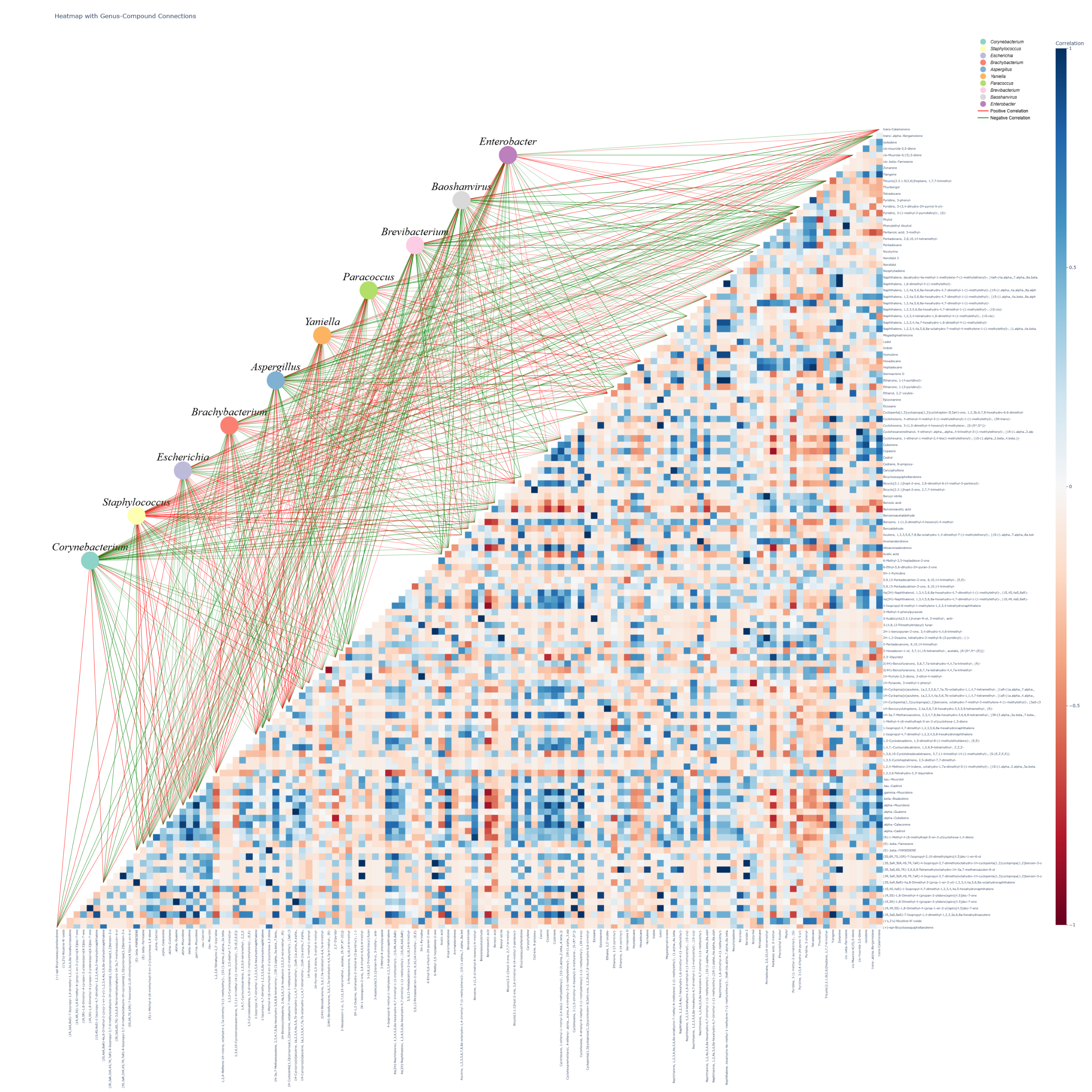


**Supplementary Figure 6.** The relationship between top 10 microorganism and VOCs was analyzed by heat map and network map with Spearman in YX4.


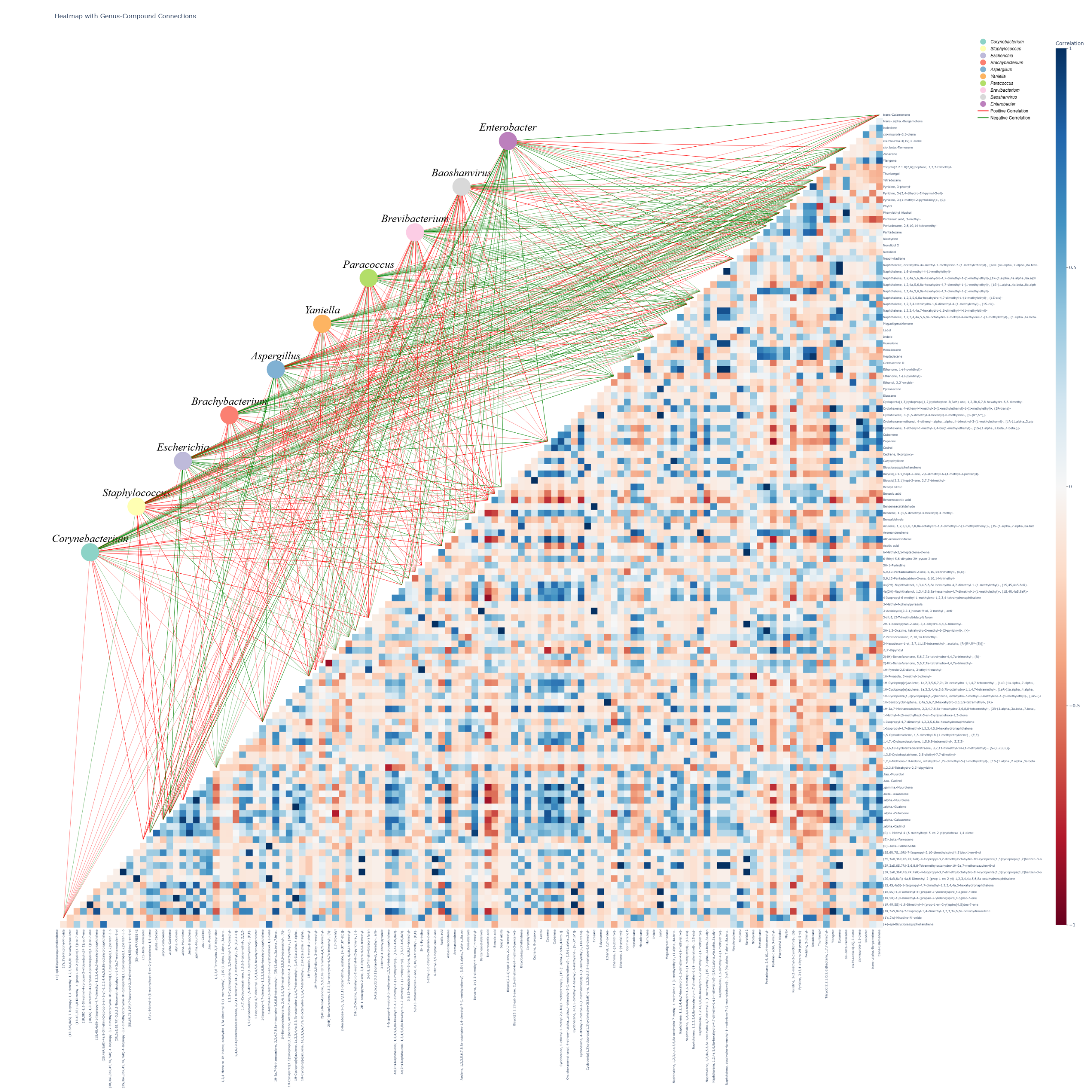
 **Supplementary Figure 7.** The relationship between top 10 microorganism and VOCs was analyzed by heat map and network map with Spearman in YX5.


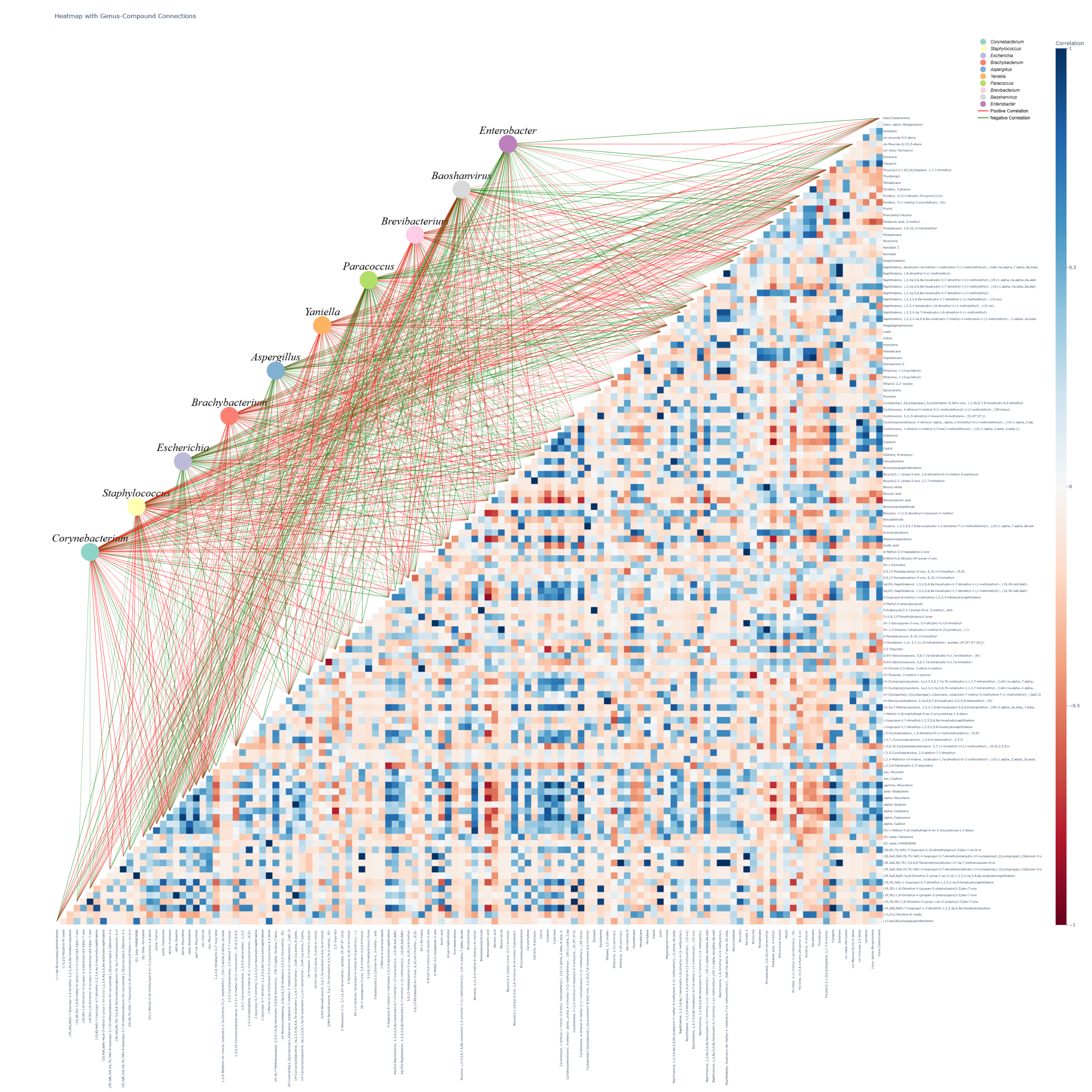


**Supplementary Figure 8.** The relationship between top 10 microorganism and VOCs was analyzed by heat map and network map with Spearman in YX6.
